# Dynamic temporal modulation of somatosensory processing during reaching

**DOI:** 10.1038/s41598-021-81156-0

**Published:** 2021-01-21

**Authors:** Dimitris Voudouris, Katja Fiehler

**Affiliations:** Department of Psychology, Justus-Liebig University Giessen, Otto-Behaghel Strasse 10F, 35394 Giessen, Germany

**Keywords:** Human behaviour, Sensorimotor processing, Sensory processing

## Abstract

Sensorimotor control of human action integrates feedforward policies that predict future body states with online sensory feedback. These predictions lead to a suppression of the associated feedback signals. Here, we examine whether somatosensory processing throughout a goal-directed movement is constantly suppressed or dynamically tuned so that online feedback processing is enhanced at critical moments of the movement. Participants reached towards their other hand in the absence of visual input and detected a probing tactile stimulus on their moving or static hand. Somatosensory processing on the moving hand was dynamically tuned over the time course of reaching, being hampered in early and late stages of the movement, but, interestingly, recovering around the time of maximal speed. This novel finding of temporal somatosensory tuning was further corroborated in a second experiment, in which larger movement amplitudes shifted the absolute time of maximal speed later in the movement. We further show that the release from suppression on the moving limb was temporally coupled with enhanced somatosensory processing on the target hand. We discuss these results in the context of optimal feedforward control and suggest that somatosensory processing is dynamically tuned during the time course of reaching by enhancing sensory processing at critical moments of the movement.

## Introduction

To plan and control a goal-directed movement, the position of the target and of the moving limb need to be known, which requires the processing and synthesis of the available sensory input. Sensory feedback processing is, however, challenged by inherent noise in the nervous system that induces uncertainty in the afferent signals^1^, which can impede movement performance^2^. In addition, sensory signals are transmitted with inevitable delays^3,4^. To compensate for these limitations, it is proposed that humans use a hybrid strategy for movement control by synthesizing sensory feedback with feedforward models that predict future sensory states based on prior knowledge of the prevailing dynamics^5^. These predictions are critical to compensate for sensorimotor noise and delays, as has also been described computationally^6,7^.

The predictions of upcoming sensory states are based on the current sensory state, estimated by sensory feedback, and on an efference copy of the motor command related to the upcoming movement^8^. When predictive and sensory feedback signals temporally and spatially match, the predicted feedback signals are suppressed. This phenomenon of predictive sensory suppression has been systematically shown in the tactile domain, e.g. during self-touch^9–11^. Interestingly, also externally generated tactile stimuli applied on a moving limb are suppressed^12–15^. This suppression also occurs on a limb that is about to move but not yet moving, for instance when a movement is planned^16,17^, planned but not executed^18,19^ or just imagined^20^, arguing for a central predictive component that generally suppresses sensory processing on that limb. The extent to which external stimuli are suppressed is used as a proxy for the processing of somatosensory signals from the stimulated body part, and therefore as a measure for the strength of the established sensorimotor predictions (see ^21^ for a review).

The strength of suppression on a moving limb is more pronounced when sensorimotor predictions are more reliable^17,22–24^. For example, when grasping an object of predictable dynamics, somatosensory suppression on the grasping hand is stronger compared to when the object’s dynamics are unknown^24^. This suggests that in the presence of reliable sensorimotor predictions, there is less need to utilize the noisy and delayed somatosensory feedback from the moving limb, and therefore afferents arising from that limb are suppressed. Because predictive and feedback signals are synthesized during a movement^4^, the way in which somatosensory feedback is used throughout the movement may influence the temporal modulation of suppression. For instance, suppression is stronger in early than late phases of a grasping movement^25,26^ (but also see ^27^), suggesting a stronger reliance on predictive control early in the movement, which is reduced in the later phase, possibly because then somatosensory feedback processing becomes more important to carefully guide the hand on the target object.

Evidently, somatosensory suppression depends on the relative reliance on predictive and feedback signals. Feedback signal processing can vary with time^28^ and recent theories of motor control, such as the optimal feedback control framework, propose that sensory feedback is temporally modulated to satisfy the goals of the ongoing action^7,29^. According to this framework, sensory feedback is combined with estimations of a forward model to first achieve an optimal estimation of the system’s state. Subsequently, a controller tunes the feedback gains to optimize motor performance, for instance by up-regulating positional feedback processing around the time of maximal reaching speed, and with down-regulating it later in the movement^30^. Accordingly, feedback processing is enhanced around the time of maximal speed, and hampered as the effector approaches the target^30,31^. Enhanced feedback gains at the time of maximal speed may reflect the importance of the reach guiding phase that begins at that moment^32^ while the reduced positional gains toward the end of the action may reflect the limitations imposed by sensorimotor delays for the processing of new sensory input before the ongoing movement is finished. Following the two assumptions of the forward model, it is unclear whether and how such temporal modulation of sensory feedback as suggested by an optimal feedback controller^30^ influences somatosensory suppression. It can be assumed that somatosensory suppression should be weakest around the time of maximal movement speed, when sensory feedback processing is greatest, and should become more pronounced as the hand approaches the target, when reliance on sensory feedback decreases.

We tested this hypothesis by asking participants to reach towards their other, unseen hand in the absence of other visual input. Somatosensory feedback processing was probed by presenting a vibrotactile stimulus of various amplitudes on the moving index finger at different moments throughout the reaching movement. After the end of the reach, participants responded whether they had felt the probing stimulus or not, a method that has been commonly applied to estimate the relative utilization of predictive and feedback signals^17,24,25^. In two experiments, we demonstrate that somatosensory processing on the moving limb is tuned throughout the reaching movement, in line with our hypothesis: Suppression is eliminated, and thus feedback processing is enhanced, around the time of maximal speed, while suppression increases gradually as the hand approaches the target. In a third experiment, we extend these findings by showing that somatosensory processing is enhanced also on the target hand, again around the moment when the reaching hand has its maximal speed. Our findings suggest an online relay of feedforward and feedback signals, which is flexibly and dynamically tuned throughout the execution of a reaching movement, linking somatosensory suppression with the temporal dynamics of sensory processing during reaching.

## Materials and methods

### Participants

Forty-eight healthy adults voluntarily joined the study; each of them participated in only one of the three experiments (Experiment 1: 19–35 years old, mean age: 24.7 years, 8 females; Experiment 2: 20–35 years old, mean age: 25.5 years, 9 females; Experiment 3: 20–34 years old, mean age: 24.9 years, 10 females). Participants were all right-handed according to the German translation of the Edinburgh Handedness Inventory^33^ (Experiment 1: 79 ± 21; Experiment 2: 94 ± 10; Experiment 3: 89 ± 13). All 48 participants gave their written informed consent prior to their participation and received course credits or 8€/hour as a compensation for their effort. Each of the three experiments was approved by the ethics committee of the Justus Liebig University Giessen and was in accordance with the Declaration of Helsinki (2013).

### Experimental setup and apparatus

**Figure 1:**
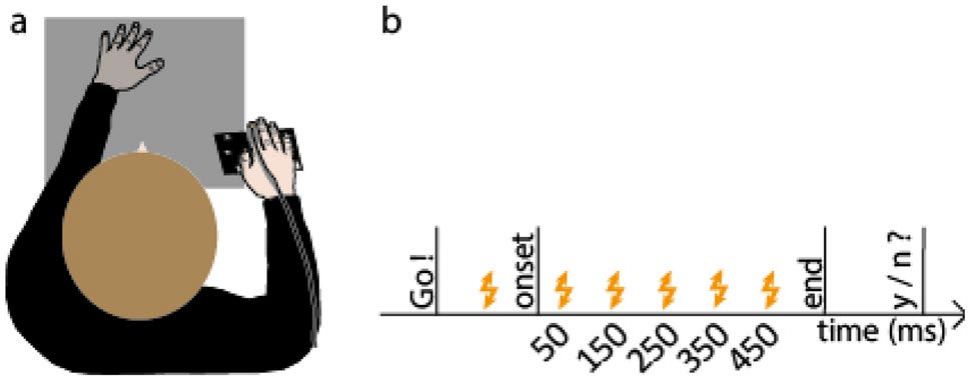
(**a**) Top view of setup and (**b**) trial schedule of Experiments 1 and 3. Participants started a right-hand reaching movement after a Go-cue toward their unseen left thumb or index finger. A brief vibrotactile probing stimulus was presented on their right index finger (Experiments 1 and 2; illustrated here) or their left little finger (Experiment 3) at several moments during the trial (thunderbolts in panel (**b**), here times are shown for Experiments 1 and 3). After the end of the reach, participants responded as to whether or not they detected the probing stimulus.

Participants sat in a dark room, in front of a table with their head resting on a chin rest. In order to examine the modulation of somatosensory feedback signals during movement, we conducted the experiment in darkness, minimizing the use of visual information about their hands or the surroundings. Participants’ right hand was resting on a button, 20 cm in front and aligned to their right shoulder. Their left hand was resting approximately 25 cm in front of them. In Experiments 1 and 3, it was 10 cm to the left of their body midline, but in Experiment 2 it was further away, approximately 25 cm to the left of their body midline. The left thumb and index finger were placed at specified positions indicated by small felt pads. A schematic depiction of the setup and the experimental timeline is shown in Fig. 1.

Brief vibrotactile stimuli (250 Hz, 50 ms) were generated through a custom-made device (Engineer Acoustics Inc., Florida, US) and presented through a stimulator (housing: 30 × 20 mm; diameter of actuator: 5 mm). Each of these stimuli could be presented in one of several moments during each trial (details below). We used 12 stimuli of different peak-to-peak amplitudes, one of which was equal to zero (no-stimulation catch trials). In Experiments 1 and 2, the amplitudes of the 11 physical stimuli ranged between 3.2 and 98 μm in steps of 9.5 μm. The stimuli in these two experiments were presented on the dorsal surface of the middle phalanx of the right index finger. In Experiment 3, the amplitudes of the 11 physical stimuli ranged between 3.2 and 35 μm in steps of 3.2 μm and were presented on the dorsal surface of the middle phalanx of the left little finger. In addition, in every trial of Experiment 3, a long, subtle noise vibrotactile stimulus (250 Hz, 1500 ms, 12 μm) was presented at trial onset on the ventral surface of the proximal phalanx of the left little finger. We introduced this noise stimulus to increase the detection thresholds and thus to avoid detection of stimuli from the lowest range of the presented amplitudes (e.g.,^34^): Having very low baseline detection thresholds in Experiments 1 and 2 would not pose any problems, as we expected suppression and thus an increase of detection thresholds. However, it would hinder the examination of somatosensory enhancement during reaching in Experiment 3, for which the ability to detect a range of stimuli weaker than those detected in the baseline was a prerequisite.

Lastly, a small infrared marker was attached to the nail of the right index finger, the position of which was recorded at 100 Hz with an Optotrak Certus (Northern Digital, Waterloo, ON, Canada). Care was taken that the cables of the vibrotactile devices and infrared marker did not constrain the participants’ movements.

### Procedure

Participants performed two blocks of trials in each Experiment; a baseline and a reaching block. We used the baseline block to establish a baseline detection threshold for each participant. The reaching block was designed to examine how the individual detection thresholds change, if at all, throughout the reaching movements. The order of the baseline and the reaching blocks was counterbalanced across participants, with half of them completing the baseline before and the other half after the reaching block.

Each trial in the baseline block started with the participant pressing and releasing the start-button with their right hand. A tone was presented 150 ms after the button press to indicate the start of the trial. A probing stimulus could be presented on the right index finger (Experiments 1 and 2) or the left little finger (Experiment 3) at 150 ms after this tone. Participants had to provide a non-speeded response as to whether they detected a stimulus or not by pressing a pedal with their left or right foot, respectively. After responding, they could start the next trial. Each catch trial was presented 18 times, and each of the 11 physical stimuli 6 times, resulting in a total of 84 trials. The baseline block lasted ~ 5 min.

In the reaching block, participants pressed the start-button and 150 ms later they heard one of two possible Go-cues that instructed a reach to their left thumb (400 Hz, 50 ms) or left index finger (800 Hz, 50 ms). Participants were instructed to reach as accurately as possible to the target finger utilizing the available somatosensory signals from their target and moving hands. No other movement instructions or feedback was given. In every trial of Experiments 1 and 3, the probing stimulus was presented at one of six different moments during the trial: one of these moments was before movement onset (100 ms after the Go-cue) and the other five moments were always during movement execution (50, 150, 250, 350, or 450 ms after movement onset as this was determined by the release of the start-button). In every trial of Experiment 2, the stimulus was presented at one of five different moments only during movement execution (50, 250, 450, 650, or 850 ms after movement onset). After each reaching movement, participants gave a non-speeded response indicating whether they had felt a probing stimulus on their right moving index finger (Experiments 1 and 2) or on the dorsal surface of their left static little finger (Experiment 3). For each moment of stimulation, each catch trial was presented 18 times, and each of the other 11 stimuli was presented 6 times, resulting in a total of 84 trials per probing moment. Therefore, at each probing moment during reaching we presented exactly the same number of 84 stimuli as those presented in the baseline. In the reaching blocks, we presented a total of 504 (Experiments 1 and 3, 6 moments of stimulation) or 420 (Experiment 2, 5 moments of stimulation) trials, each lasting ~ 35 min.

### Analysis

We first examined the kinematic behavior. To do so, we calculated the three-dimensional position of the right index finger in each reaching trial. Movement speed was calculated by means of numerical differentiation. For our offline kinematic analyses, we determined reach onset as the sample at the moment of the start-button release. This way, temporal aspects of kinematic behavior with respect to movement onset, such as the moment of maximal speed, can be directly related to the probing moments, because both are defined with respect to the same event (start-button release). Reaction time was defined as the time difference between reach onset and the onset of the Go-cue. We calculated reaction times in Experiments 1 and 3 to determine when the probing stimulus before the Go-cue was presented relative to movement onset, but not in Experiment 2 when we probed only during movement execution. The end of the movement was determined with the Multiple Sources of Information method^35^: the right index finger had to be to the left of the participant’s midline and the moment of the maximal speed had to be passed. The likelihood of a frame being the end of the movement increased with lower vertical positions, with lower speeds, and with higher tangential accelerations (i.e., there was a clear deceleration peak at the moment of contact). The moment when the product of these likelihoods was greatest was considered to be the end of the movement. We also calculated the movement time as the time difference between the onset and the end of the movement, as well as the maximal speed and the time of maximal speed relative to the total movement time.

To examine somatosensory perception, we fitted each participant’s responses to a logistic function with the maximum-likelihood estimation using the function *psignifit*^36^ in Matlab (fixed *γ* and *λ* of 0.01). This was done separately for the baseline and for each probing moment during reaching. The 50% point of the psychometric functions, defined as the detection threshold, reflects how strong the amplitude (peak-to-peak displacement) of the probing tactile stimulus had to be in order to be detected. For each participant, we subtracted their baseline detection threshold from their respective value at each probing moment in the reaching condition. This resulted in one *threshold difference* value for each probing moment for each participant, which reflects the extent to which somatosensory processing is modulated during reaching, normalized for each participant’s baseline somatosensory processing. Higher positive and lower negative threshold difference values indicate greater suppression and greater enhancement of somatosensory processing during reaching, respectively.

After calculating the threshold difference for all probing moments of each participant, we examined whether somatosensory processing was dynamically tuned during the movement according to our hypothesis. To this end, we fitted each participant’s *threshold differences* during movement with a quadratic function and contrasted its explanatory power with that provided by a simple linear function. In addition, for each participant, we calculated the time-point relative to movement onset when each of these functions had its minimum value, and we consider this time-point as the moment when somatosensory processing is mostly enhanced (or least suppressed).

After calculating all *threshold difference*s of each participant, we averaged these values for each probing moment across participants. We also calculated the minimum of each (linear and quadratic) function for each participant and then averaged these minima across participants. The average reaction time, movement time, maximal speed and relative time to maximal speed were calculated across each participant’s trials and were then averaged across participants. To examine whether somatosensory processing during reaching changed with respect to baseline, we tested whether the *threshold difference* for each probing moment was different from zero by using one-sample t-tests (one-sided, Bonferroni-Holm corrected). To investigate whether somatosensory processing changed throughout the movement, we conducted one-way repeated measures ANOVA with the factor “probing moment” on threshold differences.

Lastly, we examined whether any temporal modulation of somatosensory processing during reaching could be caused by a response bias. For instance, lower detection thresholds during reaching may stem from increased false positive responses resulting in a shift of the detection thresholds. To address this, we first calculated each participant’s baseline false-alarm rate and then subtracted this value from their respective false-alarm rate of each of the probing moments during reaching. This resulted in one *false-alarm difference* value for each probing moment for each participant, similarly to our main variable *threshold difference*. We then evaluated whether the false-alarm rates were modulated during reaching by conducting one-way repeated measures ANOVA with the factor “probing moment” on false-alarm differences.

## Results

In the first experiment, we tested the hypothesis that the expected somatosensory suppression on the reaching hand^9,13^ is temporarily modulated during reaching. This modulation would follow the pattern of sensory feedback gain processing according to an optimal feedback controller^7,30^: Suppression should be weakest, or even canceled, around the time of maximal movement speed and it should increase as the moving hand approaches the target.

We first quantified participants’ kinematic behavior. The average onset latency of the reaching movement was 591 ± 218 ms (standard deviation across participants). Participants took 503 ± 56 ms to complete the reach, their maximal speed was 95 ± 17 cm/s and it occurred at 38 ± 5% of the total movement time (on average 190 ms after movement onset).

**Figure 2:**
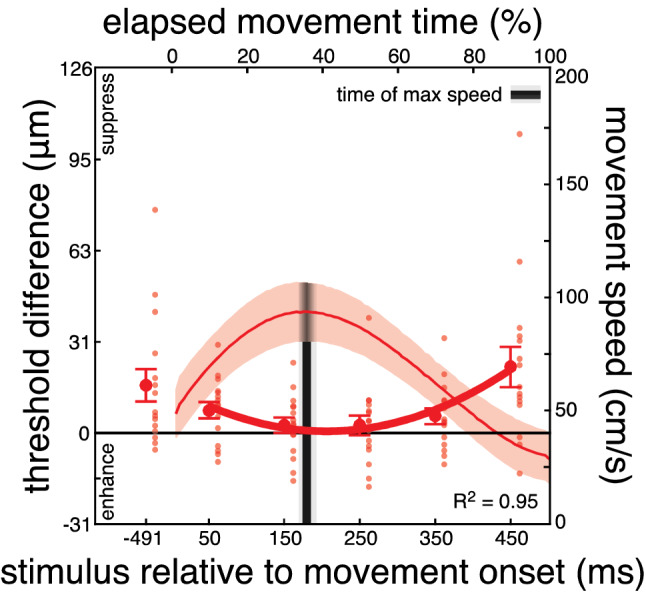
Results of Experiment 1. Differences in detection thresholds between baseline and reaching blocks across all 16 participants for each probing moment relative to movement onset (lower x-axis and left y-axis). Positive values along the left y-axis represent somatosensory suppression during movement, while zero represents no difference from baseline. Somatosensory processing is hampered on most times during the trial. However, it is similar to baseline around the time of maximal speed, moment which is illustrated with the black vertical line (gradient showing SD across participants). Large symbols show average values across participants along with their standard error and small dots represent individual participants. The thick red line represents a quadratic function fit to the participants’ average data. The time-normalized averaged movement speed is superimposed (upper x-axis and right y-axis), with standard deviation across participants (red shaded area).

Psychometric functions of all participants and conditions are available as Supplementary Material. On average, baseline detection thresholds were 13 μm (range 2.8 – 55 μm), in line with previous work^37^. During reaching, somatosensory processing was systematically hampered at all probing moments, except for those 150 ms and 250 ms after movement onset (Fig. 2; before: *t*_*15*_ = 2.95, *p* = 0.005; 50 ms: *t*_*15*_ = 2.78, *p* = 0.007; 150 ms: *t*_*15*_ = 1.01, *p* = 0.165; 250 ms: *t*_*15*_ = 0.76, *p* = 0.230; 350 ms: *t*_*15*_ = 2.13, *p* = 0.025; 450 ms: *t*_*15*_ = 3.27, *p* = 0.002). This was further supported by a main effect of probing moment on threshold differences (*F*_*5, 75*_ = 6.01, *p* < 0.001, η^2^ = 0.28). Thus, in line with our hypothesis, somatosensory suppression during movement appears to be temporally tuned.

To examine this temporal modulation in more detail, we fit each participant’s difference thresholds to a quadratic function and, as a comparison, to a simpler linear function. The quadratic function explains the individual data substantially better than the linear function (linear R^2^: 0.29 ± 0.26; quadratic R^2^: 0.77 ± 0.27, all individual fits are available in Supplementary Fig. 1), providing further support for a dynamic, temporal modulation of somatosensory processing during reaching. This result was further confirmed by a trend analysis on the group data that were well fit with a quadratic model (*F*_*1, 15*_ = 15.38, *p* = 0.001, η^2^ = 0.55), but not with a linear model (*F*_*1, 15*_ = 0.29, *p* = 0.59, η^2^ = 0.02). This is particularly evident in the observed U-shaped pattern of somatosensory modulation: suppression is clearly present in the early part of the movement (probing time of 50 ms in Fig. 2), recovers around the time of maximal speed (probing times of 150 and 250 ms), before it is gradually increasing again and reaching its maximum as the hand approaches the target (probing times of 250, 350 and 450 ms).

We then calculated for each participant the moment when the quadratic function had its minimum value with respect to movement onset, which reflects the moment at which somatosensory processing is least hampered, or even enhanced, during movement. This minimum occurred 141 ± 97 ms after movement onset, only 49 ms before the average moment of maximal speed (190 ± 39 ms after movement onset). These two moments were not different from each other (*t*_*15*_ = 2.1, *p* = 0.051; Fig. 5).

The results of Experiment 1 suggest a dynamic modulation of somatosensory processing during movement that results from a release from suppression around the time of maximal speed and from an increase in suppression as the hand approaches the target.

This dynamic tuning of somatosensory processing during movement was further supported by the results of Experiment 2. Here, a new sample of participants performed reaching movements comparable to those performed in Experiment 1, with the main difference being that the required movement amplitude was now larger, as the target hand was further away. This change led to increased movement times, and as a consequence to a later absolute time of maximal reaching speeds. Somatosensory processing was now probed on five different moments only during reaching (50, 250, 450, 650, or 850 ms after movement onset). Based on the results of Experiment 1, we hypothesize that a shift of the absolute time of maximal speed should lead to a corresponding shift of the time when somatosensory suppression should be canceled on the moving hand.

Movement times in Experiment 2 were 832 ± 157 ms. Due to the longer movement amplitudes, maximal speed was greater, at 161 ± 27 cm/s. The relative time of maximal speed occurred at 34 ± 7% with respect to the total movement time, which corresponded to 282 ± 75 ms after movement onset.

**Figure 3:**
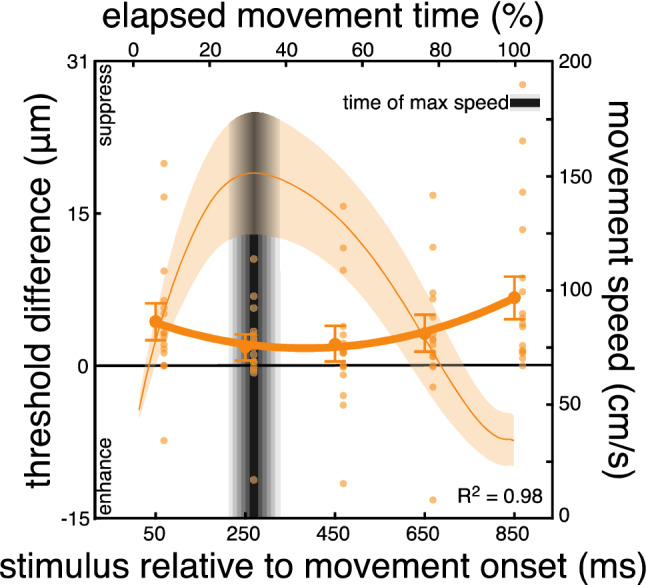
Results of Experiment 2. Differences in detection thresholds between baseline and reaching blocks. Somatosensory processing is hampered early and late in the movement, but it recovers around the time of maximal speed. As expected, the maximal speed is now much higher than in Experiment 1 due to the larger movement amplitude (compare the movement speed values along the right y-axes between Figs. 2 and 3). Details as in Fig. 2.

Baseline detection thresholds were on average 8 μm (range: 3-16 μm). Somatosensory processing during movement, as reflected in the threshold differences, was again hampered early and late during the reach, but recovered 250 ms and 450 ms after movement onset (Fig. 3; 50 ms: *t*_*15*_ = 2.88, *p* = 0.005; 250 ms: *t*_*15*_ = 1.70, *p* = 0.055; 450 ms: *t*_*15*_ = 1.42, *p* = 0.088; 650 ms: *t*_*15*_ = 1.81, *p* = 0.046; 850 ms: *t*_*15*_ = 2.53, *p* = 0.011). This was further supported by a main effect of probing moment (*F*_*4, 60*_ = 3.91, *p* = 0.048, η^2^ = 0.21).

In line with Experiment 1, somatosensory modulation was better explained by a quadratic than a linear function (linear R^2^: 0.15 ± 0.14; quadratic R^2^: 0.61 ± 0.33; all fits available in Supplementary Fig. 2). This was further confirmed by a trend analysis revealing that the group data were well fit with a quadratic function (*F*_*1, 15*_ = 5.21, *p* = 0.045, η^2^ = 0.26), but not with a linear function (*F*_*1, 15*_ = 3.3, *p* = 0.09, η^2^ = 0.19). The pattern is similar to that observed in Experiment 1: Suppression is high in the early phase of the movement (probing time of 50 ms in Fig. 3), recovers around the time of maximal speed (probing times of 250 and 450 ms), before gradually increasing as the moving hand approaches the target (probing times of 450, 650 and 850 ms).

The minimum of each participant’s quadratic fit occurred on average 310 ± 251 ms after movement onset and 28 ms after the moment of the maximal speed (282 ± 69 ms after movement onset). These two moments were not different from each other (*t*_*15*_ = −0.04, *p* = 0.68; Fig. 5). Thus, a shift of the absolute time of maximal speed led to a corresponding shift of the time of release from suppression. The results of Experiment 2 confirm and extend those of Experiment 1 and support the notion that somatosensory suppression is temporally tuned during reaching, as it is canceled around the moment of maximal speed and gradually increases as the hand approaches the target.

The results of Experiments 1 and 2 reveal two key findings. First, the processing of somatosensory afferents during reaching is neither constantly suppressed nor unaffected, but it is rather dynamically tuned throughout the movement. Second, this dynamic tuning emerges from a recovery of predictive suppression to baseline performance around the time of maximal speed and from an increase in suppression toward the end of the reach. This temporal tuning is in line with the idea that feedback gains increase around the time of maximal speed and they decrease as the hand approaches the target^30^. The recovered feedback processing around the time of maximal speed presumably denotes the need for enhanced somatosensory processing at the beginning of the guiding, deceleration phase of the movement. Thus, the combined results of Experiments 1 and 2 suggest that feedforward and feedback operations for movement control are dynamically regulated throughout the movement, in line with the assumptions of the optimal feedback control framework^30^.

Somatosensory suppression on the moving limb happens in parallel with somatosensory enhancement on the other unseen hand^13,34^. This enhancement is evident only when that hand serves as reach target and thus is relevant for the movement, but not when reaching to an external target^13^. This enhancement has low spatial resolution, as it does occur across the entire target hand irrespective to which of the fingers the reaching movement is directed to^34^. Previous work has suggested that somatosensory enhancement reflects prioritized processing of sensory signals from the target hand in order to facilitate the planning and control of the reaching movement to that hand^37^. Considering this interaction of somatosensory signals between moving and target hand, we hypothesize that the expected somatosensory enhancement on the unseen target hand (cf,^13,34^) may be temporally modulated in a similar manner as the somatosensory suppression on the moving hand as demonstrated in Experiments 1 and 2.

To examine this, we conducted a third experiment, in which we asked 16 new participants to perform the very same task as in Experiment 1, except that now the probing stimuli were presented on the little finger of their target hand. As somatosensory enhancement has low spatial resolution across the target hand^34^, we presented stimuli on a non-target digit to avoid possible suppression of these stimuli due to the expected self-generated touch caused by the reaching hand (e.g.,^22^).

Participants’ mean reach latency was 648 ± 372 ms and their movement time was 602 ± 85 ms. Maximal speed was 94.5 ± 19.4 cm/s and occurred at 33 ± 6% of the total movement time, 169 ± 36 ms after movement onset.

**Figure 4:**
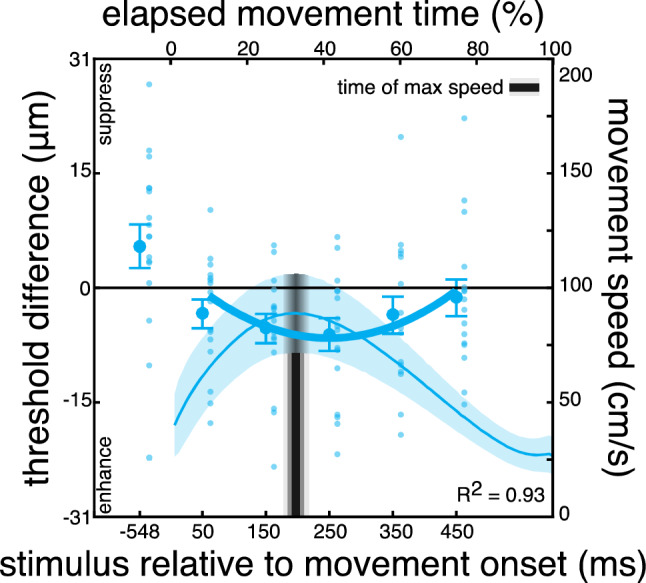
Results of Experiment 3. Differences in detection thresholds between baseline and reaching blocks on the static, target hand. Somatosensory sensitivity is generally improved during movement, but this was systematic only around the time of maximal speed of the moving hand. Details as in Fig. 2.

Baseline detection thresholds were slightly elevated compared to Experiments 1 and 2 (17 μm, range: 2–39 μm) because of the noise stimulus presented on the target digit (see Methods). Consistent with previous findings^13,34^, we found that somatosensory processing was enhanced on the target hand during the reach. Interestingly, this enhancement was systematic only 150 ms and 250 ms after movement onset (Fig. 4; before: *t*_*15*_ = 1.19, *p* = 0.074; 50 ms: *t*_*15*_ = -1.72, *p* = 0.104; 150 ms: *t*_*15*_ = -2.66, *p* = 0.018; 250 ms: *t*_*15*_ = -2.72, *p* = 0.016; 350 ms: *t*_*15*_ = -1.37, *p* = 0.190; 450 ms: *t*_*15*_ = -0.51, *p* = 0.621). This modulation was also reflected in a main effect of the probing moment (*F*_*5, 75*_ = 9.71, *p* = 0.002, η^2^ = 0.41), arguing in favor of dynamic somatosensory processing also on the static, target hand. The pattern is similar to those observed in Experiments 1 and 2: Somatosensory processing is somewhat improved early in the movement (probing time of 50 in Fig. 4), becomes systematically enhanced around the time of the reaching hand’s maximal speed (probing times of 150 and 250 ms), and gradually returns to baseline performance as the target hand is approached by the moving hand (probing times of 350 and 450 ms).

**Figure 5:**
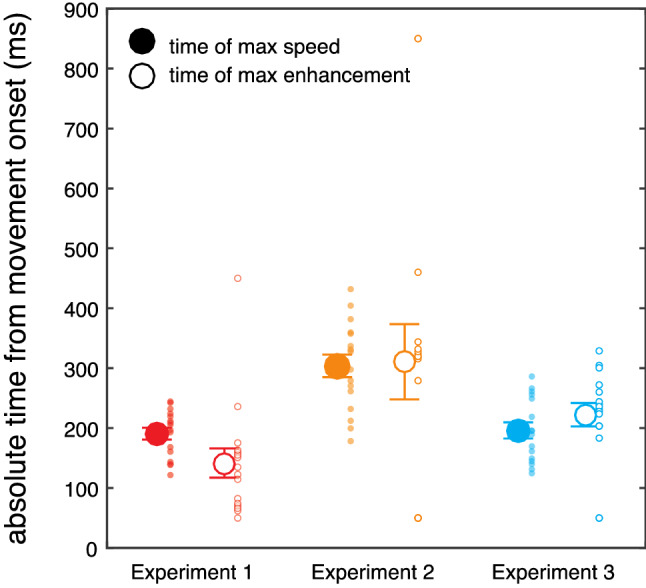
Time of absolute maximal speed and of maximal sensory enhancement for each experiment. The moment of maximal somatosensory enhancement is in close temporal proximity with the moment of maximal speed. This is evident in all three experiments. Large symbols represent averages with error bars indicating the standard error of the participants’ mean and small symbols represent individual participants.

To investigate the temporal dynamics of somatosensory enhancement in more detail, we examined whether this somatosensory modulation followed a constant or dynamic pattern. Consistent with the results of the other two experiments, a quadratic function could explain the individual modulation clearly better than a simpler linear function (linear R^2^: 0.21 ± 0.24; quadratic R^2^: 0.66 ± 0.29; all fits available in Supplementary Fig. 3). In further support of this, a trend analysis showed that the group data were well fit with a quadratic function (*F*_*1, 15*_ = 15.92, *p* = 0.001, η^2^ = 0.51), but not with a linear function (*F*_*1, 15*_ = 2.56, *p* = 0.13, η^2^ = 0.14), emphasizing that somatosensory enhancement on the target hand is indeed dynamically tuned during reaching. The average minimum of the individual quadratic fits occurred 222 ± 78 ms after movement onset, which was only 53 ms after the moment of maximal speed of the reaching hand (169 ± 36 ms). These two times were not different from each other (*t*_*15*_ = −1.1, *p* = 0.28; Fig. 5). These results support the idea that somatosensory processing is dynamically regulated also on the unseen hand that serves as reach goal, with somatosensory processing being systematically enhanced around the time of the reaching hand’s maximal speed.

All in all, we show that somatosensory processing during somatosensory-guided reaching movements is temporally modulated on both the moving and static, target hand. In Experiments 1 and 2, we show release from suppression on the moving hand around the time of maximal speed, which is concurrent with somatosensory enhancement on the static, target limb, shown in Experiment 3. These suggest that somatosensory processing on both hands is dynamically tuned during reaching, with enhanced somatosensory processing at around the same moment during the movement.

**Figure 6:**
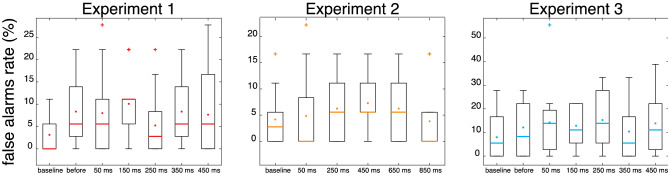
Boxplots with false-alarm rates for baseline and each probing moment in each experiment. The graphs show medians (colored lines), averages (colored dots in the box), inter-quartile range (box), most extreme data point that are not considered outliers (whiskers) and outliers (crosses).

Finally, we examined whether the temporal modulation of somatosensory processing could be affected by a change in response bias, for instance due to increased false-alarm rates at specific probing moments resulting in lower detection thresholds. We observed no main effect of probing moment on the false-alarm differences in Experiment 1 (*F*_*1, 15*_ = 1.08, *p* = 0.37, *η*^2^ = 0.06), Experiment 2 (*F*_*1, 15*_ = 1.45, *p* = 0.22, *η*^2^ = 0.08), or Experiment 3 (*F*_*1, 15*_ = 0.66, *p* = 0.65, *η*^2^ = 0.04; Fig. 6). We also found no relationship between higher false-alarm rates and enhanced somatosensory processing at any of the probing moments of Experiment 1 (all *r* < 0.43, all *p* > 0.12), Experiment 2 (all *r* < 0.20, all *p* > 0.47), or Experiment 3 (all *r* < 0.28, all *p* > 0.31). These results suggest that the temporal modulation of somatosensory processing in all three experiments is unlikely to be caused by a response bias.

## Discussion

Sensorimotor predictions generated by feedforward commands lead to suppression of the predicted afferent feedback that arises from a moving body part^22^. The strength of suppression is supposed to reflect the reliance on the established predictions: Predictions that are more reliable than the noisy sensory feedback lead to an overall lower weighting of somatosensory afferences, and thus to the phenomenon of somatosensory suppression^17,22,24^. In this study, we measured the processing of somatosensory signals on the moving limb during somatosensory-guided reaching and demonstrate that somatosensory processing is not an all-or-nothing process, i.e. being either constantly suppressed or unaffected, but that it is temporally tuned throughout the movement, expanding previous work^25,26^. Here, we demonstrate that this tuning arises from release from suppression around the time of maximal speed and from increased suppression as the hand gradually approaches the target. We further extend these results by demonstrating similar somatosensory modulation also on the static, target limb, with enhanced processing at around the moment when the reaching hand has its maximal speed. Our results suggest a flexible modulation of somatosensory sensitivity on both the moving and the target limb, presumably by synthesizing downstream motor commands with online somatosensory afferences.

Our results provide a better comprehension of somatosensory processing during feedforward and feedback control in a twofold way. First, they expand our understanding of the interplay between predictive control and sensory feedback processing during goal-directed movements. We show that somatosensory processing is dynamically modulated over the temporal evolution of somatosensory-guided reaching movements, extending assumptions of the forward model framework. The temporal modulation of somatosensory suppression is in line with the idea that humans combine predictive and feedback signals throughout the movement^5^. Our results can be explained within the optimal feedback control framework^7,30^, which suggests that feedback signals increase their gains midway of the movement and decrease their gains as the hand approaches the target. Accordingly, we demonstrate reduced suppression around the time of maximal speed, which gradually increases as the hand approaches the target (see rising curve in Figs. 2 and 3). The decrease in suppression around the time of maximal speed may arise from greater reliance on feedback signals around the time of maximal speed, which is when the reach deceleration phase begins and the movement is fine-tuned toward the target^32,38^. The increase in suppression at the time when the hand is very close to the target may result from a lower reliance on somatosensory feedback, possibly due to inherent sensorimotor delays that hinder the processing of new sensory input before the movement is finished^39^. Our results further extend previous work on the temporal modulation of visuomotor feedback gains^31^ and generalize findings of stronger suppression toward the end of visually-guided grasping^27,40^ and reaching^41^ movements to somatosensory-guided reaching actions in the absence of visual input.

Second, our results expand the well-established framework of feedforward-related somatosensory suppression^9,16,17^. We suggest that the interplay between movement-induced somatosensory predictions and corresponding feedback signals is dynamically regulated during movement. This may involve operations related to the cerebellum and somatosensory areas: The cerebellum is a key area in predictive control^5,42^, where generated predictive signals may need to be coordinated with regulatory feedback signals, particularly from somatosensory areas, considering the requirements of our task. Indeed, functional connections between the cerebellum and somatosensory areas has been recently reported in the context of somatosensory suppression^43^. In addition, close connections between somatosensory and motor areas can foster interactions between upstream somatosensory afferences and downstream motor commands. This may further contribute to the modulation of suppression, possibly by down-regulating activity in somatosensory areas through efferent signals from the supplementary motor area^18^. A critical aspect of the studies that examined the neural representation of suppression is their sole focus on discrete movements, such as force production^43,44^ or self-tickling^9^. In these cases, somatosensory feedback processing is fundamentally different than when performing a somatosensory-guided reaching movement, where somatosensory signals are essential throughout the movement. Our behavioral results suggest an *online* relay of predictive and feedback signals *during* goal-directed movements, providing behavioral support to the suggested connections found on the neural level.

In addition, we show that the time-locked release from suppression on the moving hand is complemented by concurrently enhanced somatosensory processing on the target hand. Utilizing somatosensory signals from the target hand, and thus enhancing the processing of such signals, can be particularly important for our task. First, it is well established that humans rely on somatosensory signals from both their moving and target hands during somatosensory-guided reaching^45^. In addition, tactile input from the unseen target hand improves reaching performance^46,47^. Here we show that somatosensory enhancement varies in strength throughout the movement, presumably to facilitate movement guidance to the target hand.

Movement speed may be a critical factor in determining how strongly sensory processing is modulated^48^. Faster movements can lead to stronger suppression of cortical responses to somatosensory stimuli on a moving limb^48^, possibly due to faster discharge rates in the primary motor cortex^49^ and increased afferent transmission^50^. Based on these, one might have expected stronger suppression at moments when movement speed was high. Instead, we show that suppression was absent around those moments (Figs. 2 and 3). It was also generally weaker in Experiment 2 where we observed faster movements than in Experiment 1. This supports recent findings showing that the strength of afferent input does not seem to influence the strength of suppression^51^. Rather, weaker suppression with higher speeds may be related to challenged estimations of the moving limb’s state, for instance due to increased motor noise arising from stronger motor commands^52^. In this case, enhanced sensory processing may compensate for unreliable state estimations. In support of this, muscle spindles conveying afferent input are activated more strongly with higher speeds^50^. Our results extend these findings by showing enhanced somatosensory processing at critical moments during reaching and weaker suppression with faster movements, which might be caused by a greater reliance on feedback signals.

The suppression we observed before movement onset is unlikely to be caused by feedforward motor commands, as somatosensory processing was probed considerably longer (~ 500 ms) before the time window during which suppression has been reported (~ 150–300 ms before movement onset^16,25^). This is further supported by the fact that somatosensory processing before movement onset was hampered also on the static, target hand (Experiment 3), in which case no motor command associated with a movement of that hand should have been established. The suppression of the probing stimulus during movement planning might result from the concurrent processing of the auditory Go-cue that indicated the target digit, which was presented in close temporal proximity (100 ms) to the probing stimulus.

The stronger suppression toward the end of the movement reflects weaker reliance on feedback signals at this stage, as sensorimotor delays can hinder the timely utilization of somatosensory feedback for the ongoing action. This is particularly relevant in our study because the latest probing stimuli were presented in close temporal proximity to the movement end (~ 55 ms in Experiment 1; ~ 13 ms in Experiment 2) so that any somatosensory information for movement guidance at that moment would be obsolete due to sensorimotor delays. Yet, we cannot exclude that the increased suppression toward the end of the movement may also be influenced by the expected contact between the moving and the target hand^9,41^.

Suppression on the moving hand was weakest, and thus feedback processing was greatest, at around the time of maximal reaching speed. At the same moment, somatosensory enhancement on the target hand was strongest. It is noteworthy that both the moment of maximal sensory enhancement and maximal reaching speed were calculated relative to movement onset for each participant separately, which accounts for between-participants variability in the moment of maximal speed. The moment of maximal reaching speed denotes the onset of the reach deceleration phase, which is considered to be the guiding part of the movement. Indeed, this phase is prolonged when sensory noise levels are increased^53^ or when endpoint accuracy becomes important^32,38^. Thus, humans may utilize and enhance relevant somatosensory signals at around the time of maximal movement speed in order to carefully tailor and guide their hand to the target. Therefore, the enhanced somatosensory processing on both the moving and target hands that we demonstrate around the time of maximal speed may functionally support enhanced feedback processing at the onset of the reach guiding phase.

Previous studies examined the temporal modulation of tactile suppression during visually-guided grasping movements^25,26^ and reported the opposite pattern than what we report here, namely stronger suppression in early than later phases of a movement^25,26^ (but see also^27^). We could speculate that weaker suppression toward the end of a grasping movement (e.g.,^25^) may reflect increased reliance on somatosensory feedback in order to successfully place the hand and manipulate the target object, which may not be the case in our study, where successful hand placement can be monitored also by signals from the target hand. Thus, the apparent inconsistency of the results may be due to different needs in somatosensory processing caused by specific movement demands. This is in line with our idea that suppression stems from an interplay between feedback and feedforward signals. Reliability of feedback information (visual or proprioceptive) may also modulate suppression. Despite the fact that our experiments were conducted in a dark room, it is possible that participants had some visual input due to dark adaptation, which may have influenced the strength of suppression^26^, but importantly, this is unlikely to have influenced the temporal modulation that we demonstrated here. Future work should further investigate the role of the reliability of sensory feedback on somatosensory suppression.

Feedforward sensorimotor control is considered to follow optimality principles^7,54^. From a sensory processing perspective, it is important that humans optimize and reduce the noise levels in order to access the most reliable sensory input^55,56^. From a motor control perspective, optimality can be considered as a reduction in variability while allowing the necessary flexibility to account for online changes in open-loop controlled movements^30^, possibly by tuning feedback gains by also considering the time that remains until arriving at the target^57^. To generate an optimal movement, it is necessary to estimate the state of the system so that the relevant parameters can be optimized. Internal state estimates are thus very powerful. Forward control may initially appear as a paradox, as the associated suppression of afferent signals may appear as posing complexities to ongoing actions, especially those that rely solely on somatosensory information, as in the present study. However, we show that online somatosensory processing is not be compromised at certain moments during the movement, possibly due to an active, flexible weighting of feedforward and feedback signals. This is in line with work in the context of the optimal feedback control framework that shows temporal regulation of feedback processing during visuomotor tasks^31^, with enhanced processing around the time of maximal speed and hampered sensory feedback as the hand approaches the target^30^. Our results provide novel insights into the temporal tuning of somatosensory suppression, supporting the idea that feedforward and feedback control in both the moving and the target hand is modulated across the course of a goal-directed reaching.

## Conclusion

It is well established that somatosensory signals are suppressed on a moving limb. Here, we show that such suppression can fully recover for a short period of time during a reaching movement. This release from suppression on the moving hand occurs simultaneously with enhanced somatosensory sensitivity on the unseen, target hand. This time-locked somatosensory tuning around the onset of the maximal reach speed suggests a close temporal coupling of somatosensory processing between limbs. Importantly, it further manifests that feedforward signals that cause somatosensory suppression on the moving hand are dynamically and temporally tuned based on somatosensory feedback.

## Supplementary information

Supplementary Information.

## Data Availability

The data from the reported experiments are publicly available at: https://osf.io/njw9r/.
