# Supplementary material for: Dynamic temporal modulation of somatosensory processing during reaching
Source: Sci Rep. 2021 Jan 21;11:1928. doi: 10.1038/s41598-021-81156-0 (PMC7820441; doi:10.1038/s41598-021-81156-0)
Supplement: Supplementary file 1 — Supplementary Information. [file 41598_2021_81156_MOESM1_ESM.doc]

Supplementary Material of

**Dynamic temporal modulation of somatosensory processing during reaching**

Dimitris Voudouris, Katja Fiehler

In the following Supplementary Material we provide detailed results about individual participants for each of the three experiments. More specifically, for each experiment we provide all psychometric functions of each participant, for baseline and each probing moment during reaching (Figures S1-S3). In addition, we visualize the linear and quadratic fits to the somatosensory modulation of each participant. Furthermore, we plot the individual and averaged r-squared values of each linear and quadratic fit (Figure S4) as additional evidence for the demonstrated somatosensory modulation.


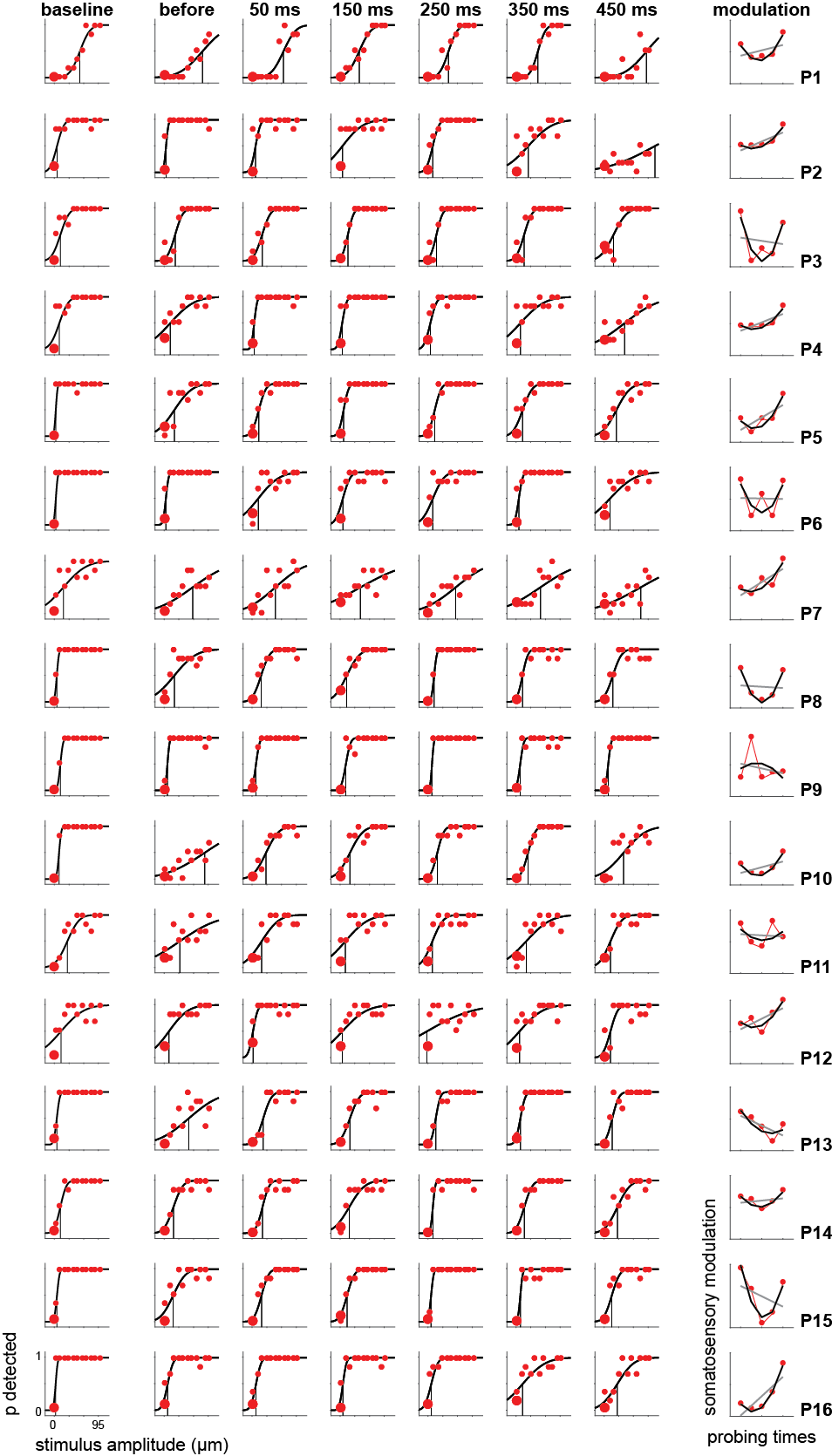


**Figure S1. Experiment 1.** Psychometric functions and somatosensory modulation for each individual participant (separate rows). The somatosensory modulation of each participant (right column) is fit with a linear (grey) and quadratic (black) function. The quadratic function fits most individual data better than the linear function.


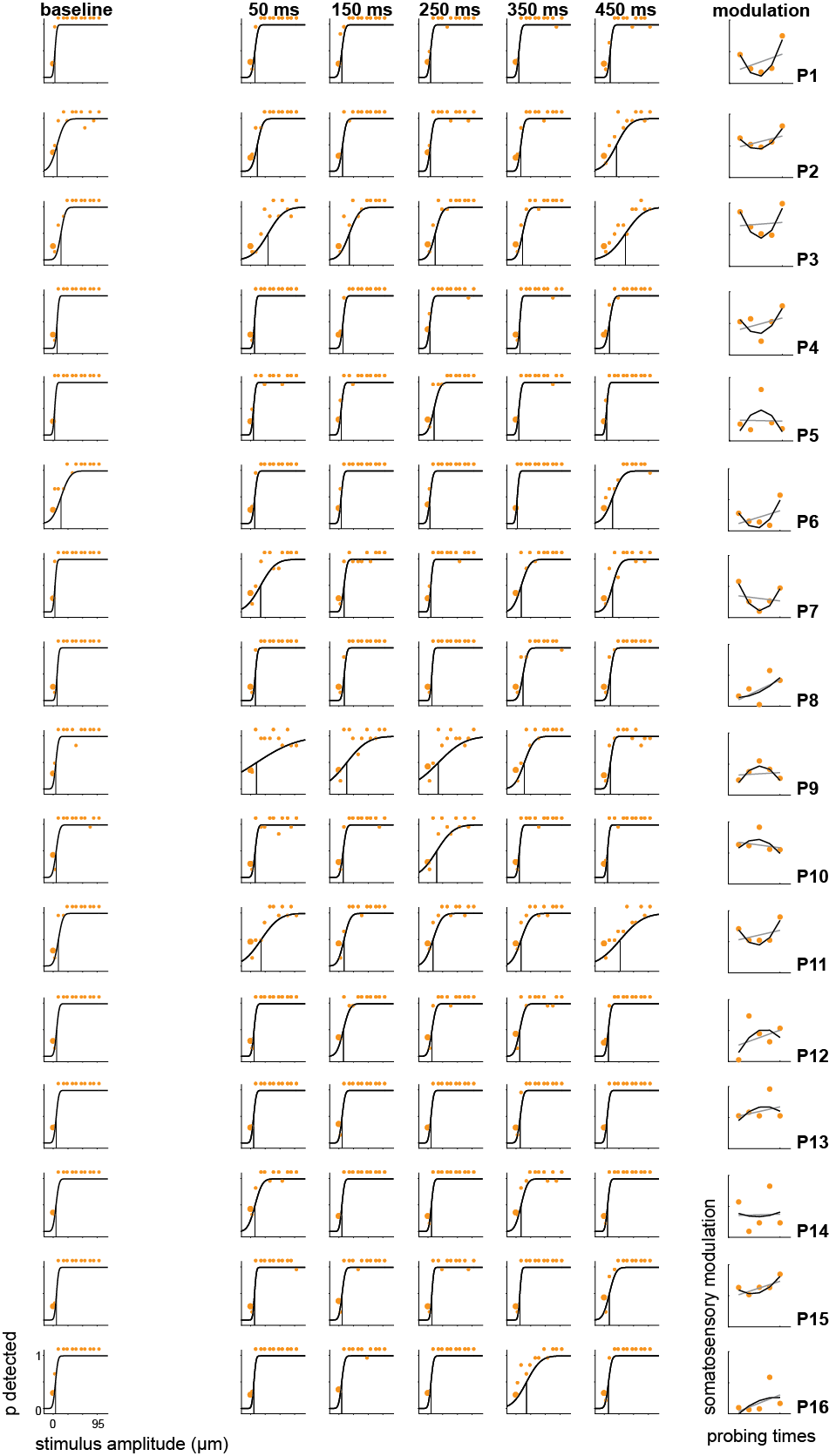


**Figure S2. Experiment 2.** Psychometric functions and somatosensory modulation for each individual participant. Details as in Figure S1.


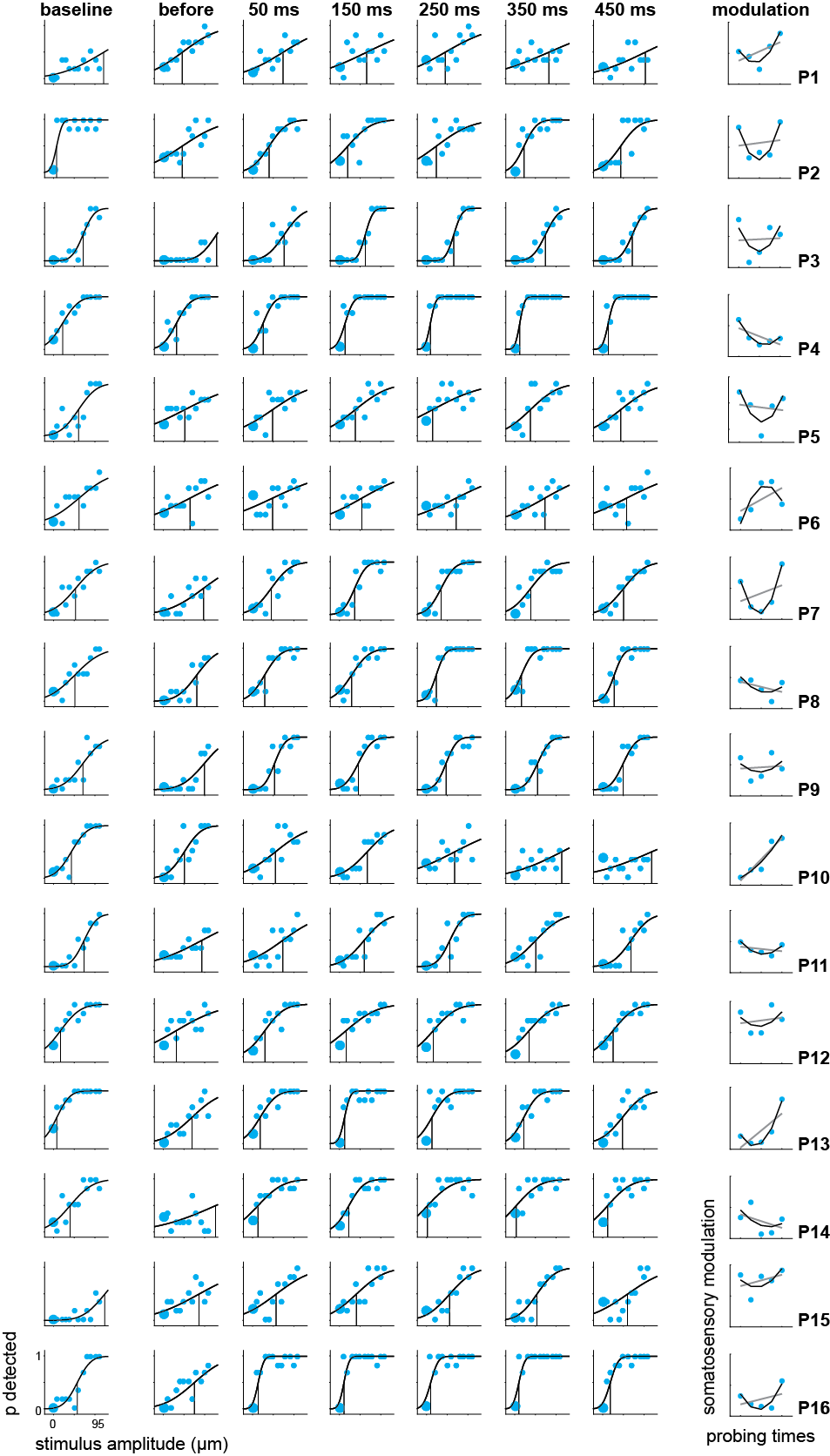


**Figure S3. Experiment 3.** Psychometric functions and somatosensory modulation for each individual participant. Details as in Figure S1.


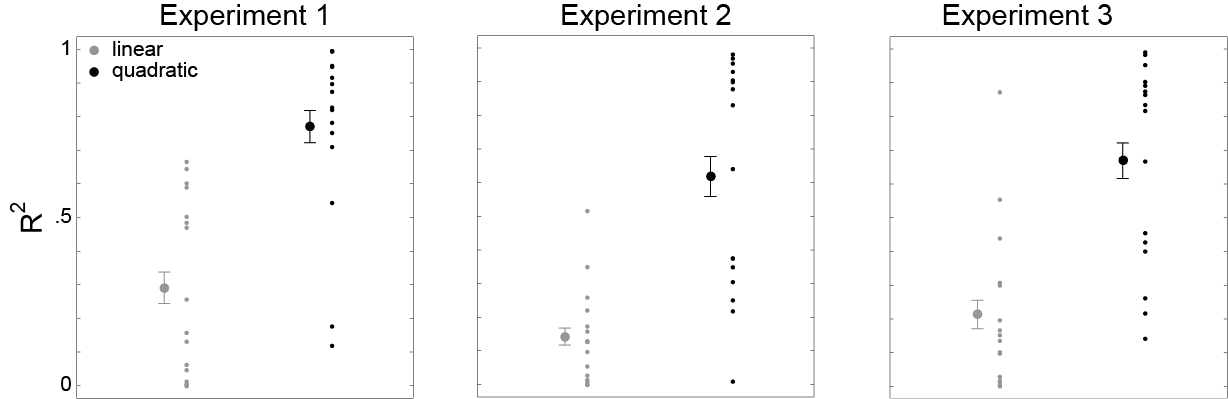
**Figure S4. Explained variance of linear and quadratic models in each experiment.** R-squared values of each linear (grey) and quadratic (black) function that was fit to individual participants (small circles), together with averages and standard error. It is evident that the quadratic fit explains more variance than the linear fit.
